# Statistical Validation of a Web-Based GIS Application and Its Applicability to Cardiovascular-Related Studies

**DOI:** 10.3390/ijerph13010002

**Published:** 2015-12-22

**Authors:** Jae Eun Lee, Jung Hye Sung, Mohamad Malouhi

**Affiliations:** 1 Research Centers in Minority Institutions Translational Research Network Data Coordinating Center, 1230 Raymond Road, Jackson, MS 39204, USA; Jung.h.lee@jsums.edu (J.H.S.); mohamad.malouhi@rtrn.net (M.M.); 2 Department of Epidemiology and Biostatistics, School of Public Health, Jackson State University, 350 W. Woodrow Wilson Drive Jackson Medical Mall, Suite 301, Jackson, MS 39213, USA

**Keywords:** Statistical validation, web-based GIS application, walkability, accessibility to healthcare facilities, density of fast-food restaurant, social determinants of cardiovascular disease

## Abstract

Purpose: There is abundant evidence that neighborhood characteristics are significantly linked to the health of the inhabitants of a given space within a given time frame. This study is to statistically validate a web-based GIS application designed to support cardiovascular-related research developed by the NIH funded Research Centers in Minority Institutions (RCMI) Translational Research Network (RTRN) Data Coordinating Center (DCC) and discuss its applicability to cardiovascular studies. Methods: Geo-referencing, geocoding and geospatial analyses were conducted for 500 randomly selected home addresses in a U.S. southeastern Metropolitan area. The correlation coefficient, factor analysis and Cronbach’s alpha (α) were estimated to quantify measures of the internal consistency, reliability and construct/criterion/discriminant validity of the cardiovascular-related geospatial variables (walk score, number of hospitals, fast food restaurants, parks and sidewalks). Results: Cronbach’s α for CVD GEOSPATIAL variables was 95.5%, implying successful internal consistency. Walk scores were significantly correlated with number of hospitals (*r* = 0.715; *p* < 0.0001), fast food restaurants (*r* = 0.729; *p* < 0.0001), parks (*r* = 0.773; *p* < 0.0001) and sidewalks (*r* = 0.648; *p* < 0.0001) within a mile from homes. It was also significantly associated with diversity index (*r* = 0.138, *p* = 0.0023), median household incomes (*r* = −0.181; *p* < 0.0001), and owner occupied rates (*r* = −0.440; *p* < 0.0001). However, its non-significant correlation was found with median age, vulnerability, unemployment rate, labor force, and population growth rate. Conclusion: Our data demonstrates that geospatial data generated by the web-based application were internally consistent and demonstrated satisfactory validity. Therefore, the GIS application may be useful to apply to cardiovascular-related studies aimed to investigate potential impact of geospatial factors on diseases and/or the long-term effect of clinical trials.

## 1. Background

Abundant evidence supports that neighborhood characteristics (e.g., walkability, accessibility to healthcare facilities, density of fast-food restaurants) have significant associations with the health of the inhabitants of a given space within a given timeframe. Chronic diseases, such as cardiovascular disease (CVD), require frequent use of healthcare services, especially comprehensive and personalized services [1]. Geographical access to healthcare facilities, therefore, is an important factor in the effective management of and continuity of care for CVD. Geographic access affects the utilization of medical care services and quality of care, influencing the patient—provider relationship, patient satisfaction, and the patient’s health status [2,3]. The negative effects of low levels of physical activity and high levels of fast-food consumption on the risk of obesity are well documented. High density of fast-food outlets has been found to have a significant association with the risk of chronic disease over time [4,5,6]. Studies have reported that measures of the community-based exercise environment, such as walkability, parks, and trails, are significant predictors of CVD [7,8,9,10,11,12,13]. Geospatial information systems (GIS) have been confirmed to be very powerful tools for identifying geospatial factors associated with chronic diseases [14], but the adoption of GIS tools in research can be impeded by, for example, difficulty acquiring community data and limited budgets for hiring experienced GIS mappers and purchasing GIS software.

Among the emerging advances in CVD health disparities research, the mutually informative multi-level and multi-modal approach (MLMM) is frequently employed at multiple community and academic sites [15,16,17]. MLMM is an approach to resolve research problem where one-dimensional models do not adequately reflect the complexity of factors involved. It has been applied to various research and clinical treatment such as cancer treatment, obesity intervention program, and CVD disparity research. MLMM does not necessarily involve data collection from multi-sites, but the study has frequently been performed in multi-sites because multi-site, interdisciplinary collaboration is highly needed for the approach. The MLMM approach requires a holistic understanding of the complex interplay between factors, such as genetics, psychological status, behavioral patterns, the environment, and community/neighborhood structure. In this method, GIS tools have frequently been used to investigate geographic disparities of disease and to determine neighborhood factors associated with specific diseases. However, study-site differences in institutional policies for protecting patient confidentiality present a major obstacle to conducting such research in a timely, effective manner. Some study sites might be reluctant to release patients’ home addresses due to institutional patient-confidentiality policies but are willing to conduct on-site geocoding instead. Standardization of procedures across sites is a major challenge in on-site geocoding for studies of multiple sites with different confidentiality policies.

The Research Centers in Minority Institutions Translational Research Network’s (RTRN) Data Coordinating Center (DCC) has developed a web-based GIS application to achieve procedural standardization for multi-site studies [18]. Statistical validation is important to verify whether this newly developed webGIS-app can measure the proposed construct. In other words, the select CVD-related neighborhood level GIS scales should produce similar results for the same or similar constructs [19]. This reliability is especially important for accomplishing the study goal to reliably determine the neighborhood risk factors for CVD. Therefore, the aim of this study is to statistically validate the webGIS-app designed to support CVD-related research by determining the measurement properties (*i.e.*, internal consistency, construct validity, *etc.*) of the data captured by the Web GIS-app. Specifically, the study is intended to answer the following questions:

(1) Internal consistency reliability: How well does the sum score of the selected CVD-related geospatial items (CVD geospatial variables) capture the expected score in the entire domain?
(2) Construct validity: How well do the CVD geospatial variables generated by the DCC GIS application measure the same construct or idea?
(3) Criterion related validity: How well do the CVD geospatial variables correlate with neighborhood socioeconomic status (SES)?
(4) Discriminant validity: How well do CVD geospatial variables discriminate severe hypertension cases from healthy controls?

Additionally, this study is to discuss the advantages and challenges of the DCC web-based GIS application to assess its potential utility for the cardiovascular-related studies.

### Overview of the RTRN-DCC Web-Based GIS Application

The RTRN-DCC webGIS-app is an interactive, web-based geocoding tool integrated with community-based datasets and open-source technologies. This tool automates the process of geocoding address data from electronic health records, the geographical mapping of research subjects, and the identification of community-based attributes. The tool takes advantage of the mapping, geocoding, and geo-processing capabilities of freely available data/system providers, including the United States Census Bureau, Yahoo, Google, MapQuest, Microsoft, Smart WalkScore, and ArcGIS. The functions of the system are carried out using distinct client-side and server-side software agents. To address data sharing, privacy, and security concerns, client-side scripting technologies were used to develop the core functions of the software. The client-side scripting architecture allows processing and executing data on the user machine, not the server.

**Figure 1:**
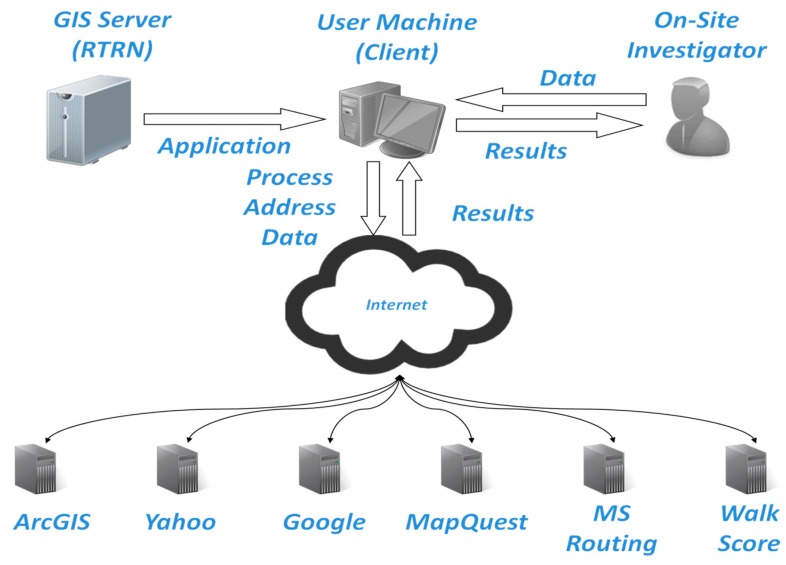
Architectural design of the GIS application.

Two elements are needed to design a GIS application, GIS functions such as the geometric services of ArcGIS and GIS data such as the data provided by the community services of Yahoo. Using multiple services (Yahoo, Google, and MapQuest) to query for community data provides a mechanism to measure the quality of the data provided by each service provider and acts as a quality control check for community data elements that belong to the same category, The use of three service providers for community data also diversifies the types of community data elements that can be searched and used for different projects and applications. Figure 1 depicts the data and application flow of the GIS application.

**Figure 2:**
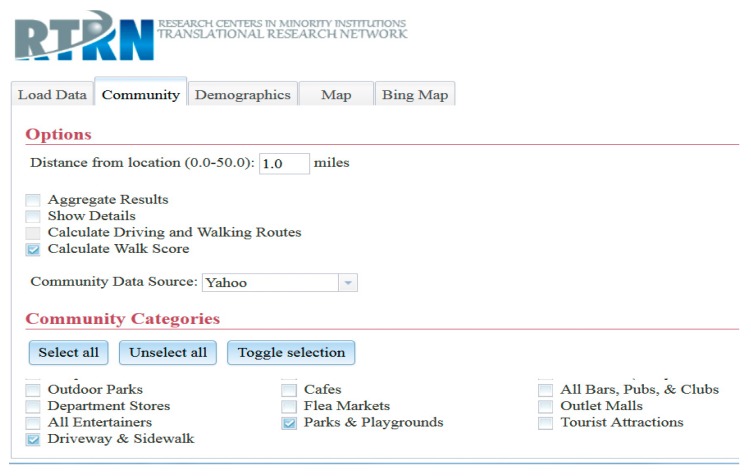
Screen capture for data input screen.

The client (user) machine has full control over the execution process and the flow of data from one component to another, so there is no need to directly transfer the processed data to the server-side application. It is used only to authenticate users, to deliver the client-side application to the user machine, and to act as a proxy for some remote services. Although not a solution for all privacy concerns, this design provides a level of protection against data being collectively intercepted by the hosting entity of the GIS tool. Aggregate and detailed community data can be retrieved from online services, and 182 different categories of community data can be searched, retrieved, and linked to each of the corresponding subjects or coordinates. Figure 2 depicts the screen capture for data input screen.

The buffered area that is created for each address point is based on an airline radius that takes into account the Earth’s surface. The application generates true geodesic buffers. Roads and topographic conditions are weighted when calculating distance between each address point and the point of an element returned by any of the community search API. The application is capable of calculating three types of distance variables: Direct airline distance, driving distance, and walking distance. The application also calculates the driving and walking times. According to many Microsoft published articles and papers, many road conditions and factors (including traffic stops) are considered when calculating both driving and walking distances.

## 2. Methods

### 2.1. Procedures for GIS Data Capturing

Geocoding and geoprocessing were conducted for 500 randomly selected home addresses in a southeastern U.S. metropolitan area. A multiphase process [20] was performed: Ensuring complete addresses, geocoding addresses to longitudinal and latitudinal coordinates, classifying and georeferencing addresses to census block groups, linking census sociodemographic characteristics to each block group, and deriving CVD-related GIS variables (walkability, accessibility to healthcare facilities) to geocoded addresses.

### 2.2. Major Neighborhood-Level Measures

*The CVD-GIS variables* were walkability, accessibility to healthcare facilities, and accessibility to fast-food restaurants. Walkability was measured by walk scores extracted from the Walk Score^®^ website (http://www.walkscore.com/) and by the number of parks and playgrounds within a mile of the house and the number of sidewalks and driveways within a mile of the house, captured from Yahoo Maps (https://maps.yahoo.com/). Driveways/sidewalks were defined as the number of lines within designated radius from home, segmented at all intersections (alley, service road, drive, street, and driveway centerline types). Walk Scores analyzed hundreds of walking routes to nearby amenities. Points are awarded based on the distance to amenities in each category: Those within a 5-min walk (0.25 miles) are given maximum points, while those within a 30-min or longer walk receive no points. Walk Scores also measure pedestrian friendliness by analyzing population density and road metrics, such as block length and intersection density. Walk Scores were found to be a valid measure of walkability [21,22,23]. Accessibility to healthcare facilities was defined as the number of hospitals or clinics within a mile of the house. Accessibility to fast-food restaurants was measured by the number of fast-food restaurants within a mile of the house. The one-mile distance from the source point is based on an airline radius that takes into account the Earth’s surface. These measures were extracted from Yahoo Maps (https://maps.yahoo.com/).

*Neighborhood Socioeconomic Status (SES) at the block-group level* was determined based on 2010 U.S. Census and Environmental Systems Research Institute (ESRI) data. The measures were median household income, unemployment rate, annual population growth rate, median age, median house value, and the percentage of owner-occupied units. Social vulnerability and diversity indices developed by ESRI were also included. Social vulnerability refers to vulnerability to natural and man-made disasters because of population and housing characteristics, such as age, low income, disability, and home value. Despite its potential for chronic disease research, only a single study has shown that social vulnerability has a significant association with chronic disease [24]. The diversity index shows the likelihood that two persons randomly chosen from the same area belong to different race or ethnic groups [25]. Selection of the SES variables was based on the literature review.

### 2.3. Statistical Analysis

Data analysis in the present study was performed using SAS version 9.3. Cronbach’s α was estimated to test the internal consistency of the CVD-related neighborhood factors. Internal consistency refers to how closely a set of CVD-related neighborhood factors is related as a group, which indicates the consistency of the scales in reflecting the construct they measure. Cronbach’s α of 0.70 and higher was set as an acceptable value for reliability scales [26].

Construct validity was tested by conducting Pearson correlation coefficient analysis to examine whether CVD-related factors were correlated with each other. Factor analysis was performed to explore the interrelationship among the CVD factors and to identify patterns among the items by highlighting their similarities and differences. Construct validity is the degree to which a test measures what it claims, or purports, to be measuring. A correlation coefficient of ≥0.50 which is defined as moderate or higher correlation [27] was considered satisfactory. If all factor loadings per item in a factor were greater than 0.5, then we concluded that the CVD-GIS items were loaded on the single factor and that the CVD-GIS items were unidimensional.

Criterion-related validity shows how much a certain CVD item (Walk Score) is correlated with another validated instrument with a similar structure (neighborhood socioeconomic factors). Correlations were assessed with Pearson’s correlation coefficients.

For discriminant validity, we drew on research results presented at the 2013 Minority Health Genomics Resource for Health Disparity Research Network Annual Meeting at the Renaissance Hotel in Atlanta, Georgia, 21–22 August 2013. The study analyzed data from 426 African-American participants in a case-control study and geospatial data captured using the RTRN-DCC webGIS-app. Discriminant validity was tested by comparing the least square means (adjusted means estimated from linear models) of each geospatial CVD risk factor in the severe hypertension and the healthy control groups. Severe hypertension was defined as controlled hypertension (SBP ≤ 140, DBP ≤ 90, taking two or more blood pressure medications) and resistant hypertension (>140/90 and taking three or more medications).

## 3. Results

*Internal Consistency*: The CVD geospatial variables derived from the RTRN DCC webGIS-app showed excellent reliability. The internal consistency of each item is shown in Table 1. The Cronbach’s α coefficient was considered satisfactory when it equaled 0.776. The correlation of each designated item with the summated score for all other items was greater than 0.7.

**Table 1:** Internal consistency for cardiovascular factors.

Cardiovacular Factors	Raw Variables		Standardized Variables	
	Correlation with Total	Alpha (0.776) *	Correlation with Total	Alpha (0.955) *
Smart Walk Score	0.732	0.868	0.754	0.964
Hospital/Clinics within a mile	0.837	0.726	0.877	0.943
Fast food Restaurants w/in a mile	0.893	0.76	0.93	0.934
Parks/play grounds within a mile	0.913	0.727	0.931	0.934
Sidewalks/driveways within a mile	0.781	0.653	0.879	0.943

* Overall Cronbach Coefficient α.

*Construct validity*: Five CVD geospatial variables had strong associations with each other (*r* for all five CVD-GIS variables ≥0.7), indicating similarity (Table 2). The factor analysis supported the hypothesized factor solution that all five items would be loaded in a single factor. All factor loadings for five items in Factor one were greater than 0.5, and Factor one explained 85% of total variance (Table 3), indicating that the five items in the CVD geospatial were unidimensional.

**Table 2:** Pearson Correlation Coefficient among cardiovascular factors.

Cardiovascular Factors	Walk Score	Hospitals	Fast-Food	Parks	Sideway
Smart Walk Score	1	0.715	0.729	0.773	0.648
		<0.0001	<0.0001	<0.0001	<0.0001
# Hospitals	0.715	1	0.89	0.847	0.792
	<0.0001		<0.0001	<0.0001	<0.0001
# Fast food Restaurants	0.729	0.89	1	0.876	0.903
	<0.0001	<0.0001		<0.0001	<0.0001
# Parks/Playgrounds	0.773	0.847	0.876	1	0.905
	<0.0001	<0.0001	<0.0001		<0.0001
# Sidewalks/Driveways	0.648	0.792	0.903	0.905	1
	<0.0001	<0.0001	<0.0001	<0.0001	

**Table 3:** Factor analysis for cardiovascular factors.

Factor Pattern	Factor 1	Factor 2	Factor 3	Factor 4	Factor 5
Smart Walk Score	0.833	0.545	0.071	0.053	0.035
Hospital/Clinics	0.923	−0.048	−0.364	−0.09	0.067
Fast food Restaurants	0.958	−0.131	−0.071	0.215	−0.116
Parks/Play grounds	0.958	−0.036	0.145	−0.223	−0.103
Sidewalks/Driveways	0.927	−0.269	0.223	0.049	0.129
Variance Explained	4.24 (85%)	0.39(8%)	0.21 (4%)	0.11 (2%)	0.05 (1%)

*Criterion validity*: Criterion validity was supported by significant correlations between CVD geospatial variables and neighborhood SES (Table 4). Walk Scores had significant correlations with the numbers of hospitals, fast-food restaurants, parks and playgrounds, and sidewalks and driveways within a mile of a home. Walk Scores were also significantly associated with the diversity index, median household income, and owner-occupied rate. Walk Scores were found to have insignificant correlations with mean age, social vulnerability, unemployment rate, labor force, and population growth rate. A similar pattern persisted between other CVD-GIS items and neighborhood SES variables (not shown in the table). Persistent pattern was also held in the correlation of SES variables with the factor scores, which represent composites of the five CVD geospatial variables. Population density was highly correlated with population growth rate (*r* = 0.62; *p* > 0.001) and there was no difference in correlation pattern with CVD geospatial variables between density and growth rate.

**Table 4:** Correlation coefficients between walk score and each of neighborhood socio-economic factors.

Neighborhood Socio-Economic Factors	Correlation Coefficients with Walk Score	*p*-Value
Annual Population Growth Rate	−0.024	0.5919
Diversity Index	0.138	0.0023
Labor Force Rate	−0.066	0.1462
Median Age	−0.018	0.6867
Median Home Value	−0.045	0.3197
Median Household Income	−0.181	<0.0001
% Owner Occupied	−0.44	<0.0001
Unemployment Rate	0.021	0.6466
Social Vulnerability	0.027	0.5552

*Discriminant Validity* Compared to the severe hypertension group, the healthy control group had significantly higher least-square mean of Walk Scores (47.9 *vs.* 39.6, *p* = 0.0045), numbers of hospitals (4.0 *vs.* 2.1, *p* = 0.0087) and numbers of fast-food restaurants (3.0 *vs.* 1.8, *p* = 0.0056), parks and playgrounds (4.6 *vs.* 2.6, *p* = 0.0011), and sidewalks and driveways (7.6 *vs.* 3.3, *p* = 0.0062) within a mile of the home. This pattern persisted even after controlling for age, marital status, employment, and education.

## 4. Discussion

Validating this web-based GIS tool designed to data-mine CVD-related environmental factors allowed assessing the reliability and validity of the data captured by the RTRN DCC webGIS-app. The present study revealed that geospatial data generated by the webGIS-app for all five CVD-GIS variables had good internal consistency. The strong internal consistency in this study (Cronbach’s α > 0.75) represented a single construct of the items, meaning that the five items had the same concept to measure the cardiovascular risk. The factor analysis results also confirmed that select CVD geospatial community factors are a single construct. The analysis revealed that all five items were loaded on the single factor, indicating that selected CVD-related geospatial variables were unidimensional. Criterion validity was also supported by the significant associations of the CVD geospatial factors with some communities’ SES, which is consistent with other studies [28,29]. Discriminant validity was supported by select CVD geospatial factors that distinguished severe hypertension cases from healthy controls, although Quereshi *et al.* failed to demonstrate a significant correlation between the age-adjusted annual incidence rates of ischemic stroke among residents in each of the 63 cities in Minnesota and average Walk Score for 63 cities [21]. Therefore, the RTRN DCC webGIS-app generated valid, reliable community data, which can be used for CVD-related research.

This study, however, did not consider random structure of the geospatial features in the analysis. The high correlation among five CVD-related geospatial variables may be produced by the high spatial autocorrelation of each variable. The non-significant correlation of each CVD geospatial variable with median age, vulnerability and other socioeconomic variables may depict a lack of variability in the sample. Indeed, our additional analysis revealed that Moran’s I for each five CVD geospatial variables were significantly non-zero, implying that there was spatial autocorrelation and lack of variability in the measures. The lack of variability and high spatial autocorrelation may be caused by our samples, which were from a specific U.S. Southern Metropolitan area. Therefore, further study is needed including samples from broader areas.

Another limitation of the study is that the estimated CVD geospatial measures were based on a great circle distance (e.g., # of hospitals within one mile from home), which may not represent the real accessibility to resources. However, our additional analysis using the data including 780 hospitals within a mile from home revealed that mean driving distance was 1.45 ± 0.66, implying the one mile that we used for estimation was within one standard deviation of the mean driving distance, thereby we cannot conclude that the great circle distance differed from driving distance. Another analysis including 1477 samples found that the difference between two distances (large circle distance and driving distance) increased with increase of great circle distance (*r* = 0.77). However, within a mile of the great circle distance, the difference between two distances was small (mean difference = 0.18 ± 0.22). Therefore, our results may not be significantly biased by the great circle distance that we used for estimating the CVD geospatial data.

The RTRN DCC GIS application offers numerous advantages. First, it is cost effective. Many studies on the impact of environmental factors on the disease have used, for example, alcohol control policy data from state departments, alcohol outlets data from city liquor license commissioners, and crime data from city police departments. However, obtaining data from these institutions might not be easy, and the data might not be real-time as the geospatial environment changes constantly. The Big Data technology used for the RTRN DCC GIS application allowed easy capturing of real-time, physical, CVD-related, social and built environmental data. Additionally, commercial GIS software (e.g., ArcGIS) is expensive and hard for the layperson to operate. However, learning to operate the RTRN DCC GIS application is easy and requires little investment in time even from the layperson. Therefore, this technology can reduce study costs.

Second, the RTRN DCC GIS application helps avoid measurement errors. Due to the difficulty of obtaining community data, studies typically have used perceived measures (e.g., alcohol outlets, crime, grocery stores), instead of physical measures. However, perceived measures might not precisely reflect the actual environment [30], and studies have reported poor agreement between perceived and objective measures of physical environment characteristics [31,32,33]. When using perceived measures, participants’ behavior can pose a possible source of error. For example, participants who drink more frequently might be more aware of the outlets in their environment and provide more accurate and probably fuller counts of them [34].

Third, the RTRN DCC GIS application is applicable to multisite clinical studies that require procedural standardization of geocoding and geoprocessing. To generate reliable, valid spatial data through onsite geocoding, standardization of procedures across sites (e.g., geocoding software, GIS data, algorithms, and methods) is necessary. The webGIS-app successfully achieved procedural standardization in a multi-site hypertension-use case study.

Fourth, DCC GIS application can incorporate diverse measures of the physical environment that are potentially related to CVD risk. For example, raw pollution data available from US Environmental Protection Agency website can be assigned into each block group by using mathematical models and assigned pollution data can be incorporated into DCC GIS system for the subsequent analyses. Distance from home address to parks and/or walking trail can be estimated using the DCC GIS app.

However, it is important to note that a few challenges can affect research validity, reliability and efficiency. Service providers (e.g., ESRI, Yahoo) might change their service policies (e.g., changing search categories, charged services) during the research period. If research takes too long and ongoing geocoding is planned, policy changes might affect the output and, thus, reliability and validity. Limited flexibility is another limitation of the GIS app. Although designed to capture relevant CVD-related data for research, it can still be applied in other fields if additional community data are included in the application (e.g., pollution data, alcohol outlets, crime rates). Another challenge might be the daily application program interface (API) quotas imposed by service providers to prevent such system traffic. These quotas might result in discontinuation of geocoding and geoprocessing. Therefore, it is important to empower the webGIS-app with Big Data technology, such as cloud computing. 

Cloud initiatives can facilitate fast, seamless client access to powerful processing services hosted at remote locations. It is noteworthy that like all GIS systems, the DCC GIS app has difficulty conducting research including participants from rural areas since many rural residents have Post Office Box mailing address. The U.S. Postal Service does not deliver mail to residents living in more rural towns whose physical addresses do not receive any form of USPS carrier delivery service. Instead, they have to obtain a P.O. Box to receive mail without a yearly fee [35].

## 5. Conclusions

The RTRN DCC web-based GIS application might be useful in CVD-related research in which short-term enrollment or retrospective geocoding is planned, and data capture is the main purpose of using the tool. This tool successfully captured geospatial data for a multi-site hypertension case-control study. This customized tool cut costs for GIS software and personnel, reduced the time needed for training and allowed standardization of procedures across sites and on-site geocoding for sites reluctant to release patient data. The RTRN DCC GIS application could also be applied to other fields of epidemiology studies to investigate the association of the community environment with diseases and to the phase IV clinical trials to determine the modifying effect of geospatial factors on trial efficacy.
